# Supplementary material for: Germination Stimulant Activity of Isothiocyanates on Phelipanche spp
Source: Plants (Basel). 2022 Feb 24;11(5):606. doi: 10.3390/plants11050606 (PMC8912868; doi:10.3390/plants11050606)
Supplement: Supplementary file 1 [file plants-11-00606-s001.zip › plants-1446934-supplementary.pdf]

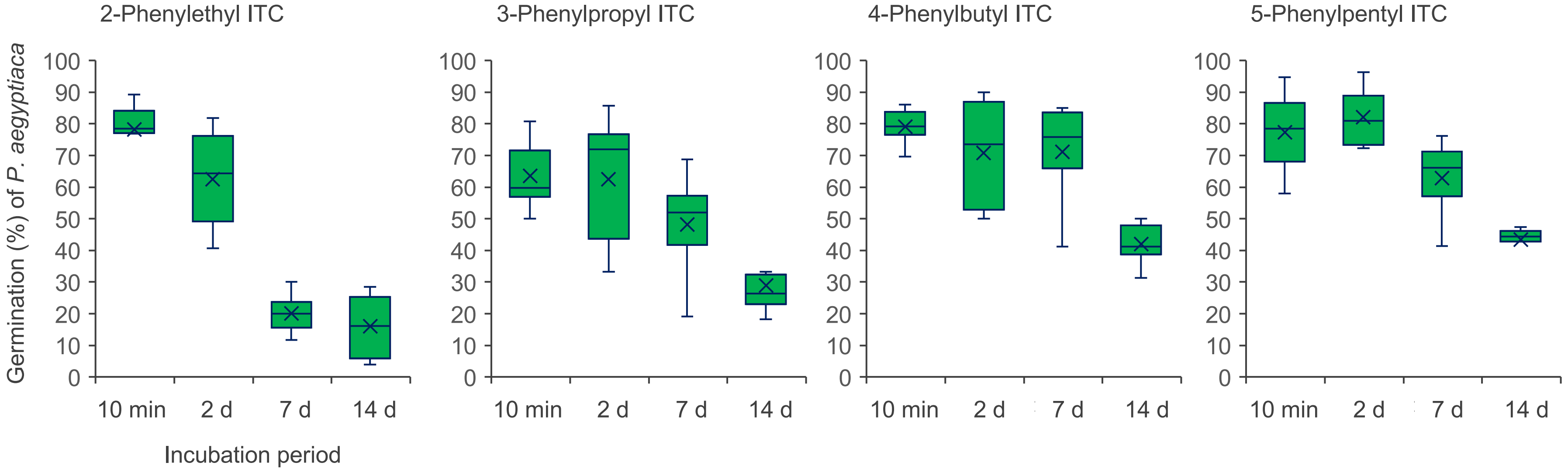

Supplementary Figure S1. Residual activity of ITCs on *P. aegyptiaca* germination. Each ITC dissolved in water (200 ml, concentration of ITC is 10^{-7} M) was applied into vermiculites (300 g) in the pot and incubated at 22 C under light/14h and dark/10h. Then, 100-200 mL water was poured into vermiculites and running water including ITCs from bottom of pots was extracted with ethyl acetate. Concentrated crude extracts were used for germination assay. The box represents the interquartile range, whiskers represent the maximum and minimum values, the middle indicates the median, and the x within the box represents the mean (n=4).

A

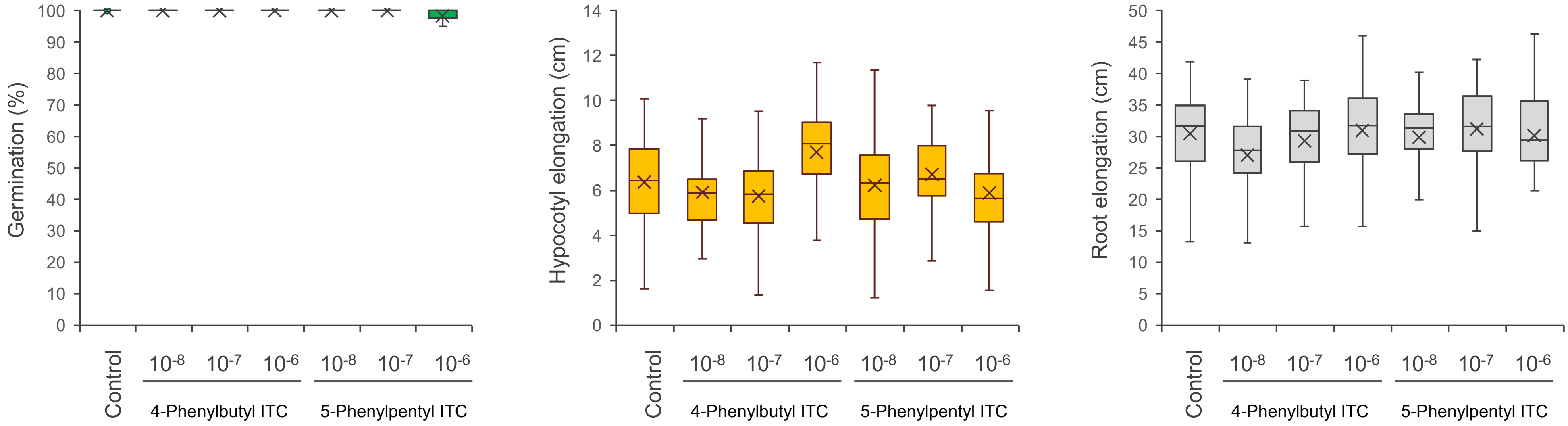

Supplementary Figure S2. Effects of ITCs on germination and growth of host plant cabbage. The box represents the interquartile range, whiskers represent the maximum and minimum values, the middle indicates the median, and the x within the box represents the mean (n=10).

[3-Phenylpropyl ITC]

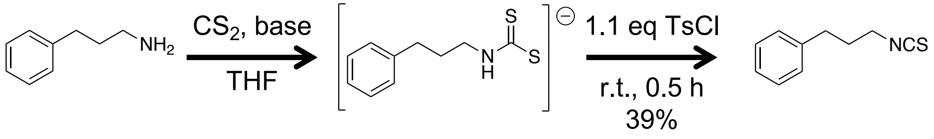

[4-Phenylbutyl ITC]

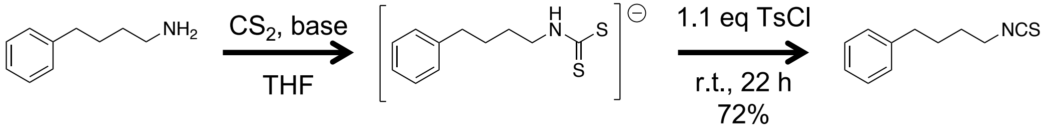

[5-Phenylpentyl ITC]

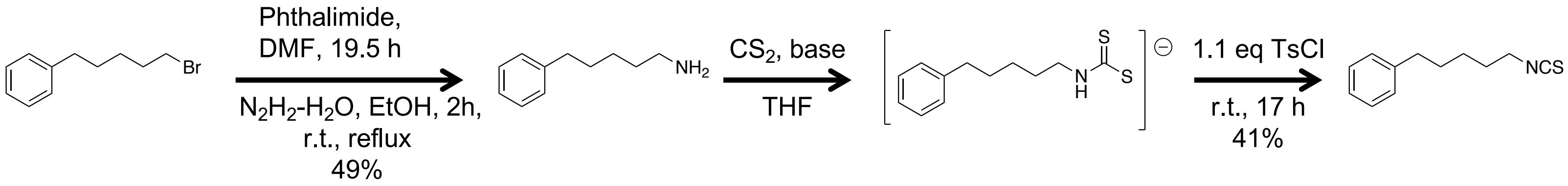

[6-Phenylhexyl ITC]

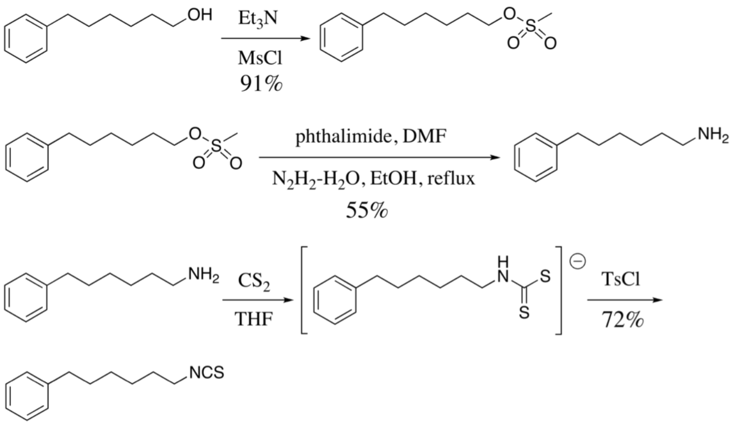

Supplementary Figure S3. Synthetic schemes of ITCs.
